# Publisher Correction: MOG analogues to explore the MCT2 pharmacophore, α-ketoglutarate biology and cellular effects of *N*-oxalylglycine

**DOI:** 10.1038/s42003-022-03922-8

**Published:** 2022-09-27

**Authors:** Louise Fets, Natalie Bevan, Patrícia M. Nunes, Sebastien Campos, Mariana Silva dos Santos, Emma Sherriff, James I. MacRae, David House, Dimitrios Anastasiou

**Affiliations:** 1 Cancer Metabolism Laboratory, The Francis Crick Institute, London, UK; 2 Crick–GSK Biomedical LinkLabs, London, UK; 3 Metabolomics Science Technology Platform, The Francis Crick Institute, London, UK; 4 Present Address: Drug Transport and Tumour Metabolism Lab, MRC London Institute of Medical Sciences, London, UK

**Keywords:** Small molecules, Metabolomics

Correction to: *Communications Biology* 10.1038/s42003-022-03805-y, published online 26 August 2022.

The original version of this Article was missing several references in the following paragraph in the Introduction:

“In addition to MOG, MCT2 transports endogenous monocarboxylates ranging from pyruvate and lactate to larger ketone bodies such as β-hydroxybutyrate, acetoacetate, α-ketoisovalerate and α-ketoisocaproate^**19**^ (Supplementary Fig. 1a) with a higher affinity than the other SLC16 family members^**9**^. MCT2 plays important physiological roles including the uptake of astrocyte-secreted lactate into neurons within the brain. MCT2 is highly expressed in some human cancers (Supplementary Fig. 1b) and has been proposed as a biomarker for prostate cancer, as well as having pro-tumorigenic and pro-metastatic roles in breast cancer.”

The *correct* version of this paragraph should appear as:

“In addition to MOG, MCT2 transports endogenous monocarboxylates ranging from pyruvate and lactate to larger ketone bodies such as β-hydroxybutyrate, acetoacetate, α-ketoisovalerate and α-ketoisocaproate^**19**^ (Supplementary Fig. 1a) with a higher affinity than the other SLC16 family members^**9**^. MCT2 plays important physiological roles including the uptake of astrocyte-secreted lactate into neurons within the brain^**20-21**^. MCT2 is highly expressed in some human cancers (Supplementary Fig. 1b) and has been proposed as a biomarker for prostate cancer^**22**^, as well as having pro-tumorigenic^**23**^ and pro-metastatic^**24**^ roles in breast cancer.”

References #20-24 were mistakenly cited in the following sentence in the Introduction:

“Here, we report the design and synthesis of MOG-based analogues and use them to explore the MCT2 pharmacophore, and [NOG]IC-dependent interference with intracellular targets in the context of their effects on cellular proliferation and survival^**20-24**^.”

The *correct* version of this sentence should appear as:

“Here, we report the design and synthesis of MOG-based analogues and use them to explore the MCT2 pharmacophore, and [NOG]IC-dependent interference with intracellular targets in the context of their effects on cellular proliferation and survival.”

Both errors have now been corrected in the PDF and HTML versions of the Article.

