# Supplementary material for: MOG analogues to explore the MCT2 pharmacophore, α-ketoglutarate biology and cellular effects of N-oxalylglycine
Source: Commun Biol. 2022 Aug 26;5:877. doi: 10.1038/s42003-022-03805-y (PMC9418262; doi:10.1038/s42003-022-03805-y)
Supplement: Supplementary file 2 — Supplementary Information [file 42003_2022_3805_MOESM2_ESM.pdf]

Synthesis of MOG Analogues

2-((2-Methoxy-2-oxoethyl)amino)-2-oxoacetic acid (MOG, 1)

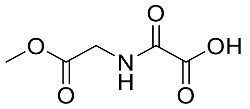

This compound was prepared according to the procedure described in Fets *et al.*, *Nat. Chem. Biol.* 2018, 14, 1032–1042.

tert-Butyl 2-chloro-2-oxoacetate (General Intermediate)

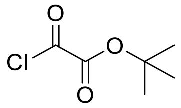

A stirred solution of oxalyl chloride (5.08 g, 40 mmol) in Et_2O (80 mL) was cooled at 0 °C and *tert*-butanol (2.97 g, 40 mmol) in Et_2O (20 mL) was added dropwise under N_2. The resulting mixture was stirred for 30 min at 0 °C and then 16 h at room temperature. The solvent was evaporated to afford *tert*-butyl 2-chloro-2-oxoacetate a colourless oil. This material was used in crude form for the preparation of each of the intermediate described below.

tert-Butyl 2-(2-ethoxy-2-oxoethylamino)-2-oxoacetate (Intermediate 2)

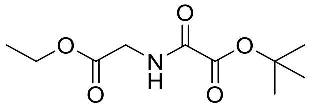

tert-Butyl 2-chloro-2-oxoacetate (712 mg, 4.32 mmol) was dissolved in CH_2Cl_2 (5 mL) and added dropwise to a solution of glycine ethyl ester hydrochloride (604 mg, 4.32 mmol) and Et_3N (874 mg, 8.65 mmol) in CH_2Cl_2 (20 mL) under N_2 at 0 °C. The resulting mixture was stirred from 0 °C to room temperature for 3 h. The solvent was evaporated under reduced pressure and the residue was purified by chromatography on silica eluting with petrol ether:EtOAc (3:1) to afford the title product (738 mg, 73%) as a clear oil. **LCMS** Rt = 1.35 min, $[2M+Na]^+$ = 485.3; **^{1}H NMR**.(400 MHz, CD_3OD) δ 4.22 (q, *J* = 8.0 Hz, 2H), 4.03 – 4.00 (m, 2H), 1.58 (s, 9H), 1.29 (t, *J* = 8.0 Hz, 3H).

2-((2-Ethoxy-2-oxoethyl)amino)-2-oxoacetic acid (2)

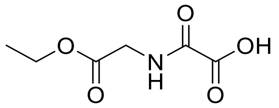

A mixture of *tert*-butyl 2-(2-ethoxy-2-oxoethylamino)-2-oxoacetate (728 mg, 3.15 mmol) and CF_3COOH (3 mL) in CH_2Cl_2 (10 mL) was stirred at room temperature for 3 h. The solvent was evaporated under reduced pressure. Et_2O was added to the residue and removed under

reduced pressure to afford the title product (388 mg, 100%) as an off-white solid. **^{1}H NMR** (400 MHz, DMSO-d$_6$) δ 9.10 (t, *J* = 6.0 Hz, 1H), 4.11 (q, *J* = 7.2 Hz, 2H), 3.87 (d, *J* = 6.0 Hz, 2H), 1.19 (t, *J*=7.2 Hz, 3H).

***tert*-Butyl 2-(2-isopropoxy-2-oxoethylamino)-2-oxoacetate (Intermediate 3)**

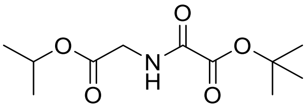

tert-Butyl 2-chloro-2-oxoacetate (1.074 g, 6.53 mmol) was dissolved in CH_2Cl_2 (10 mL) and added dropwise to a solution of glycine isopropyl ester hydrochloride (1.00 g, 6.53 mmol) and Et_3N (1.32 g, 13 mmol) in CH_2Cl_2 (20 mL) under N_2 at 0 °C. The resulting mixture was stirred from 0 °C to room temperature for 3 h. The solvent was evaporated under reduced pressure and the residue was purified by chromatography on silica eluting with petrol ether:EtOAc (3:1) to afford the title product (212 mg) as a clear oil. **LCMS** Rt = 1.43 min, $[2M+Na]^+$ = 513.3; **^{1}H NMR**.(400 MHz, $CDCl_3$) δ 7.51 (s, 1H), 5.12 (spt, *J* =6.0 Hz, 1H), 4.06 (d, *J* = 6.0 Hz, 2H), 1.59 (s, 9H), 1.30 (d, *J* = 6.0 Hz, 6H).

2-((2-Isopropoxy-2-oxoethyl)amino)-2-oxoacetic acid (3, IPOG)

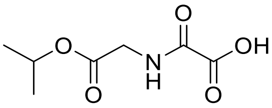

A mixture of *tert*-butyl 2-(2-isopropoxy-2-oxoethylamino)-2-oxoacetate (132 mg, 0.54 mmol) and CF_3COOH (3 mL) in CH_2Cl_2 (10 mL) was stirred at room temperature for 3 h. The solvent was evaporated under reduced pressure and the solid freeze dried to afford the title product (52 mg, 51%) as a white solid. **^{1}H NMR** (400 MHz, DMSO-d$_6$) δ = 9.07 (t, *J* = 6.0 Hz, 1H), 4.92 (spt, *J*=6.3 Hz, 1H), 3.83 (d, *J*=6.0 Hz, 2H), 1.19 (d, *J*=6.3 Hz, 6H).

(*R*)-Methyl 2-(2-(*tert*-butoxy)-2-oxoacetamido)propanoate (Intermediate 4)

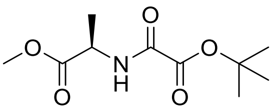

(*R*)-methyl 2-aminopropanoate hydrochloride (746 mg, 5.34 mmol) was dissolved in CH_2Cl_2 (5 mL) and Et_3N (1.08 g, 10.7 mmol) was slowly added. The mixture was stirred at 0 °C for 10 min. *tert*-Butyl 2-chloro-2-oxoacetate (800 mg, 4.86 mmol) was added dropwise and the resulting mixture was stirred from 0 °C to room temperature for 3 h. The solvent was evaporated under reduced pressure and the residue was purified by chromatography on silica eluting with petrol ether:EtOAc (3:1) to afford the title product (520 mg, 46%) as a white solid.

^{1}H NMR (400 MHz, CD_3OD) δ 4.49 (q, *J* = 8.0 Hz, 1H), 3.76 (s, 3H), 1.58 (s, 9H), 1.46 (d, *J* = 8.0 Hz, 3H).

(*R*)-Methyl 2-aminopropanoate (4)

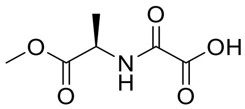

A mixture of (*R*)-methyl 2-(2-(*tert*-butoxy)-2-oxoacetamido)propanoate (740 mg, 2.24 mmol) and CF_3COOH (3 mL) in CH_2Cl_2 (10 mL) was stirred at room temperature for 3 h. The solvent was evaporated under reduced pressure and the solid was dried by lyophilization to afford the title product (560 mg, 100%) as an off-white solid. **^{1}H NMR** (400 MHz, DMSO-d6) δ 9.12 (d, *J* = 7.3 Hz, 1H), 4.34 (quin, *J* = 7.4 Hz, 1H), 3.63 (s, 3H), 1.34 (d, *J* = 7.3 Hz, 3H).

(*S*)-Methyl 2-(2-(*tert*-butoxy)-2-oxoacetamido)propanoate (Intermediate 5)

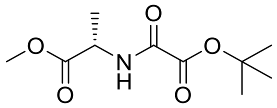

tert-Butyl 2-chloro-2-oxoacetate (368 mg, 2 mmol) in CH_2Cl_2 (2 mL) was added dropwise to a solution of (*S*)-methyl 2-aminopropanoate hydrochloride (284 mg, 2 mmol) and Et_3N (267 mg, 2.24 mmol) in CH_2Cl_2 (5 mL) at 0 °C. The resulting mixture was stirred from 0 °C to room temperature for 3 h. The solvent was evaporated under reduced pressure and the residue was purified by chromatography on silica eluting with petrol ether:EtOAc (4:1) to afford the title product (112 mg, 24%) as a colourless oil. **^{1}H NMR** (400 MHz, $CDCl_3$) δ 7.59 (s, 1H), 4.63 – 4.59 (m, 1H), 3.80 (s, 3H), 1.59 (s, 9H), 1.49 (d, *J* = 8.0 Hz, 3H).

(*S*)-Methyl 2-aminopropanoate (5)

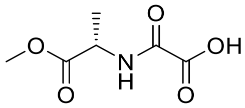

A mixture of (*S*)-methyl 2-(2-(*tert*-butoxy)-2-oxoacetamido)propanoate (112 mg, 0.48 mmol) and CF_3COOH (3 mL) in CH_2Cl_2 (10 mL) was stirred at room temperature for 3 h. The solvent was evaporated under reduced pressure to afford the title product (83 mg, 99%) as a white solid. **^{1}H NMR** (400 MHz, DMSO-d_6) δ 9.09 (d, *J* = 7.3 Hz, 1H), 4.34 (quin, *J* = 7.3 Hz, 1H), 3.64 (s, 3H), 1.34 (d, *J* = 7.3 Hz, 3H).

Methyl 2-(2-(*tert*-butoxy)-2-oxoacetamido)-2-methylpropanoate (Intermediate 6)

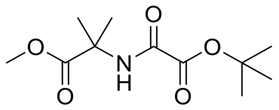

tert-Butyl 2-chloro-2-oxoacetate (591 mg, 3.6 mmol) in CH_2Cl_2 (10 mL) was added to a solution of methyl 2-amino-2-methylpropanoate (500 mg, 3.24 mmol) and Et_3N (690 mg, 6.83 mmol) in CH_2Cl_2 (20 mL) at 0 °C under a nitrogen atmosphere. The resulting mixture was stirred for 5 min at 0 °C and at room temperature for 2 h. Saturated aqueous $NaHCO_3$ (30 mL) was added, the organic phase was separated, dried over Na_2SO_4, filtered and evaporated under reduced pressure to afford the crude title product as a yellow oil (870 mg, >100%). This material was taken into deprotection step without purification.

2-((1-Methoxy-2-methyl-1-oxopropan-2-yl)amino)-2-oxoacetic acid (6)

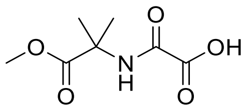

To a stirred solution of methyl 2-(2-(*tert*-butoxy)-2-oxoacetamido)-2-methylpropanoate (870 mg, 3.55 mmol) in CH_2Cl_2 (21.3 mL) was added CF_3COOH (7 mL). The reaction mixture was stirred at room temperature for 7 h. The solvent was evaporated under reduced pressure. The residue was purified by HPLC (Xbridge Prep C18 OBD column 19 x 150 mm 5 µm; Mobile Phase A: Water (0.1% CF_3COOH), Mobile Phase B: acetonitrile; flowrate 20 mL/min; gradiant 5% B to 25.6% B in 5 min; UV detection 254 nm) to afford the title product (257 mg, 24%) as a white solid after freeze drying. **LCMS** Rt = 1.07 min, $[MH]^+$ = 190.0; **^{1}H NMR** (400 MHz, DMSO-d_6) δ 13.97 (s, 1H), 8.99 (s, 1H), 3.59 (s, 3H), 1.40 (s, 6H).

***tert*-Butyl 2-oxo-2-((2-oxopropyl)amino)acetate (Intermediate 7)**

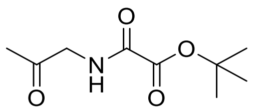

1-Aminopropan-2-one hydrochloride (431 mg, 3.93 mmol) in CH_2Cl_2 (20 mL) was cooled to 0 °C and Et_3N (795 mg, 7.86 mmol) was added. The reaction mixture was stirred at room temperature for 10 min and then cooled to 0 °C. *tert*-Butyl 2-chloro-2-oxoacetate (647 mg, 3.93 mmol) was added dropwise and the resulting mixture was stirred from 0 °C to room temperature for 3 h. The solvent was evaporated under reduced pressure and the residue was purified by chromatography on silica eluting with petrol ether:EtOAc (1:1) to afford the title product (71 mg, 9%) as a yellow oil. **LCMS** Rt = 1.22 min, $[2M+Na]^+$ = 425.3.

2-Oxo-2-((2-oxopropyl)amino)acetic acid (7)

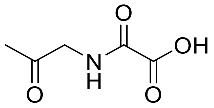

A mixture of *tert*-butyl 2-oxo-2-((2-oxopropyl)amino)acetate (71 mg, 0.35 mmol) and CF_3COOH (2 mL) in CH_2Cl_2 (6 mL) was stirred at room temperature for 3 h. The solvent was evaporated under reduced pressure to afford the title product (51 mg, 100%) as an off-white solid. **^{1}H NMR** (400 MHz, DMSO-d_6) δ 8.84 (br. s., 1H), 3.98 (d, *J* = 6.0 Hz, 2H), 2.09 (s, 3H).

***tert*-Butyl 2-((oxazol-2-ylmethyl)amino)-2-oxoacetate (Intermediate 8)**

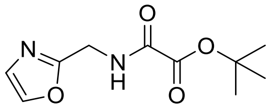

Oxazol-2-ylmethanamine hydrochloride (204 mg, 2 mmol) and Et_3N (420 mg, 4.16 mmol) in CH_2Cl_2 (20 mL) were cooled to 0 °C and stirred for 20 min. *tert*-Butyl 2-chloro-2-oxoacetate (342 mg, 2 mmol) in CH_2Cl_2 (10 mL) was added dropwise and the resulting mixture was stirred from 0 °C to room temperature for 3 h. The solvent was evaporated under reduced pressure and the residue was purified by chromatography on silica eluting with petrol ether:EtOAc (1:1) to afford the title product (187 mg, 71%) as a white solid. **^{1}H NMR** (400 MHz, $CDCl_3$) δ 7.67 (s, 2H), 7.12 (s, 1H), 4.67 (d, *J* = 6.0 Hz, 2H).

2-((Oxazol-2-ylmethyl)amino)-2-oxoacetic acid (8)

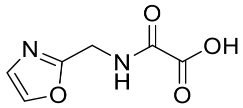

A mixture of *tert*-butyl 2-((oxazol-2-ylmethyl)amino)-2-oxoacetate (187 mg, 0.83 mmol) and CF_3COOH (3 mL) in CH_2Cl_2 (10 mL) was stirred at room temperature for 3 h. The solvent was evaporated under reduced pressure to afford the title product (135 mg, 96%) as a white solid. **^{1}H NMR** (400 MHz, DMSO-d_6) δ 9.35 (t, *J* = 6.0 Hz, 1H), 8.03 (s, 1H), 7.15 (s, 1H), 4.43 (d, *J* = 6.0 Hz, 2H).

***tert*-Butyl 2-oxo-2-(((5-(trifluoromethyl)furan-2-yl)methyl)amino)acetate (Intermediate 9)**

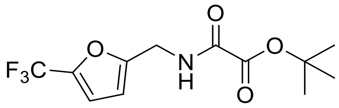

tert-Butyl 2-chloro-2-oxoacetate (134 mg, 0.82 mmol) in CH_2Cl_2 (5 mL) was added to a solution of (5-(trifluoromethyl)furan-2-yl)methanamine hydrochloride (150 mg, 0.746 mmol) and Et_3N (158 mg, 1.574 mmol) in CH_2Cl_2 (25 mL) at 0 °C under nitrogen atmosphere. The resulting mixture was stirred for 5 min at 0 °C and at room temperature for 2 h. Saturated aqueous

$NaHCO_3$ (30 mL) was added, the organic phase was separated, dried over Na_2SO_4, filtered and evaporated under reduced pressure to afford the crude title product as a white solid (567 mg, >100%). This material was taken into deprotection step without purification.

2-Oxo-2-(((5-(trifluoromethyl)furan-2-yl)methyl)amino)acetic acid (9)

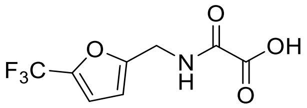

To a stirred solution of *tert*-Butyl 2-oxo-2-(((5-(trifluoromethyl)furan-2-yl)methyl)amino)acetate (567 mg, 1.935 mmol) in CH_2Cl_2 (12 mL) was added CF_3COOH (3.87 mL). The reaction mixture was stirred at room temperature for 7 h. The solvent was evaporated under reduced pressure. The residue was purified by HPLC HPLC (Xbridge Prep C18 OBD column 19 x 150 mm 5 µm; Mobile Phase A: Water (0.1% CF_3COOH), Mobile Phase B: acetonitrile; flowrate 20 mL/min; gradiant 1% B to 100% B in 3 min; UV detection 220 nm) to afford the title product (73 mg, 16%) as a white solid after freeze drying. **LCMS** Rt = 0.66 min, $[M-H]^-$ = 236.0; **^{1}H NMR** (300 MHz, DMSO-d_6) δ 13.99 (s, 1H), 9.39 (t, *J* = 5.4 Hz, 1H), 7.15-7.14 (m, 1H), 6.49-6.48 (m, 1H), 4.38 (d, *J* = 6 Hz, 2H).

***tert*-Butyl 2-(((3-methyl-1,2,4-oxadiazol-5-yl)methyl)amino)-2-oxoacetate (Intermediate 10)**

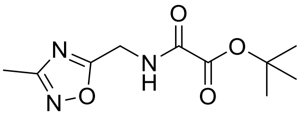

tert-Butyl 2-chloro-2-oxoacetate (182 mg, 1.11 mmol) in CH_2Cl_2 (5 mL) was added to a solution of (3-methyl-1,2,4-oxadiazol-5-yl)methanamine hydrochloride (150 mg, 1 mmol) and Et_3N (213 mg, 2.11 mmol) in CH_2Cl_2 (25 mL) at 0 °C under nitrogen atmosphere. The resulting mixture was stirred for 5 min at 0 °C and at room temperature for 2 h. Saturated aqueous $NaHCO_3$ (30 mL) was added, the organic phase was separated, dried over Na_2SO_4, filtered and evaporated under reduced pressure to afford the crude title product as a yellow oil (238 mg, 99%). This material was taken into deprotection step without purification.

2-(((3-Methyl-1,2,4-oxadiazol-5-yl)methyl)amino)-2-oxoacetic acid (10)

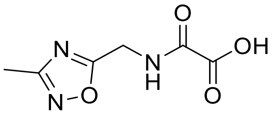

To a stirred solution of *tert*-butyl 2-(((3-methyl-1,2,4-oxadiazol-5-yl)methyl)amino)-2-oxoacetate (238 mg, 0.987 mmol) in CH_2Cl_2 (6 mL) at 0 °C under nitrogen atmosphere was added CF_3COOH (2 mL). The reaction mixture was stirred at 0 °C for 5 min and at room

temperature for 6 h. The solvent was evaporated under reduced pressure. The residue was purified by HPLC (XSelect Prep C18 OBD column 19 x 150 mm 5 µm; Mobile Phase A: Water (0.05% CF_3COOH), Mobile Phase B: acetonitrile; flowrate 20 mL/min; gradiant 2% B to 15% B in 7 min; UV detection 254 nm) to afford the title product (31 mg, 17%) as a white solid after freeze drying. **LCMS** Rt = 0.28 min, $[MH]^+$ = 185.9; **^{1}H NMR** (400 MHz, DMSO-d_6) δ 14.16 (s, 1H), 9.55 (t, *J* = 5.6 Hz, 1H), 4.57 (d, *J* = 6 Hz, 2H), 2.32 (s, 3H).

SUPPLEMENTARY FIGURES

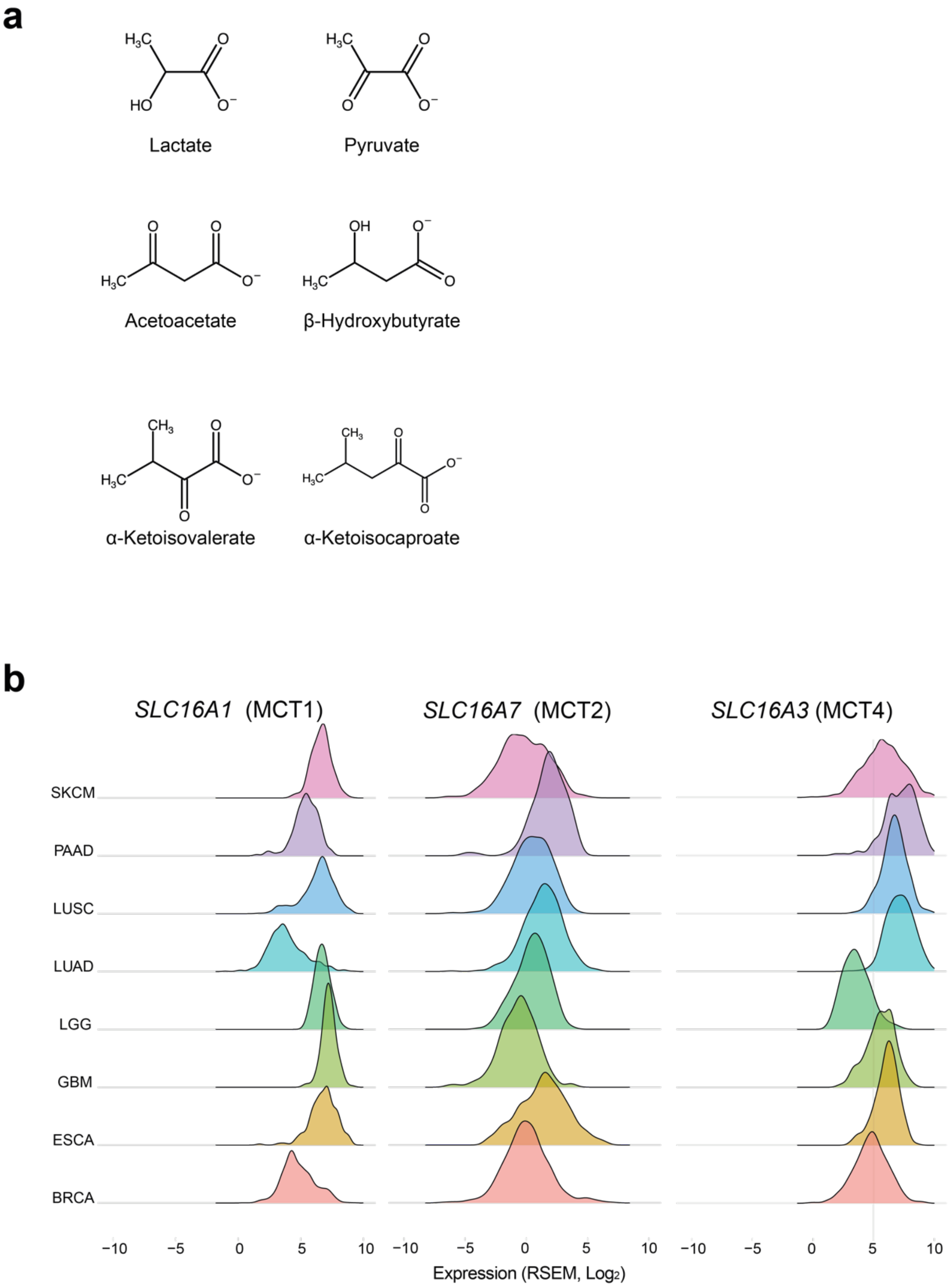

Supplementary Figure 1

a) Chemical structures of endogenous MCT2 substrates.
b) Ridge plots showing the range of expression levels of *SLC16A1* (MCT1), *SLC16A7* (MCT2) and *SLC16A3* (MCT4) in different human cancer types. Data source: The Cancer Genome Atlas, SKCM – Skin cutaneous melanoma; PAAD – Pancreatic Adenocarcinoma; LUSC – Lung squamous cell carcinoma; LUAD – Lung adenocarcinoma; LGG – Low grade glioma; GBM – Glioblastoma; ESCA – Oesophageal carcinoma; BRCA – Breast Invasive Carcinoma.

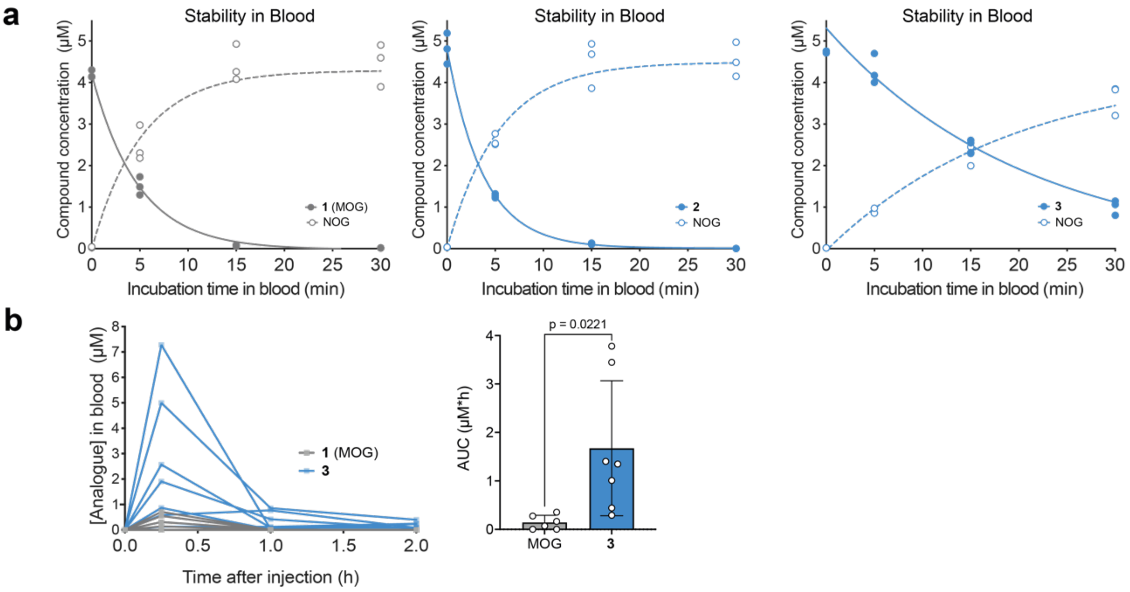

Supplementary Figure 2

a) Stability of MOG, **2** and **3** in blood over time assessed by LC-MS measurements of the levels of parent compound and NOG produced from their degradation (n=3 technical replicates, sampled at each of the indicated time points).
b) MOG or compound **3** were administered intraperitoneally to mice (MOG: n=6 mice, **3**: n=7 mice) at 100 mg/kg and concentration of the compounds in blood samples collected at different time points were quantified by LC-MS. The bar graph shows the means (±SEM) of areas under the curve values for each compound.

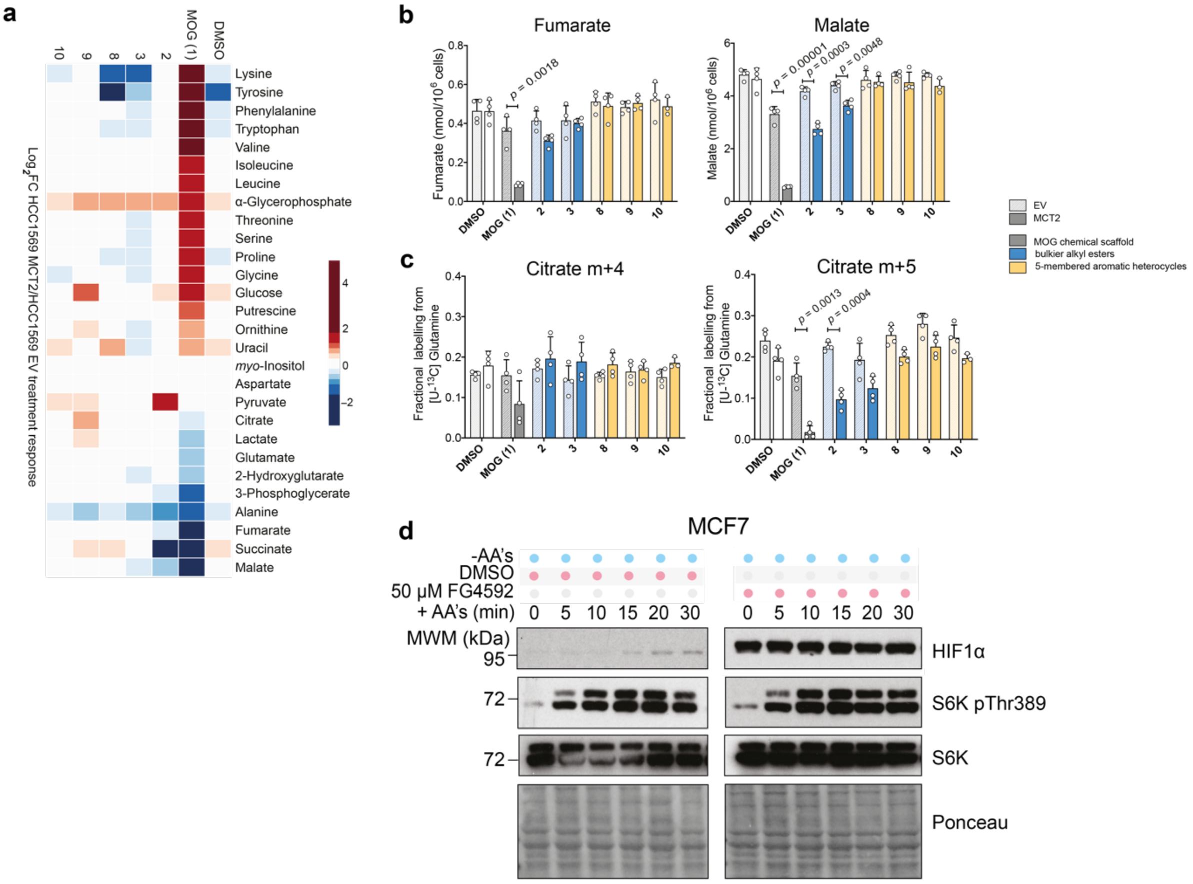

Supplementary Figure 3

a) Heat map showing $\log_2$ fold-changes in the abundance of the indicated metabolites in HCC1569-MCT2 cells relative to HCC1569-EV cells treated with DMSO (vehicle control) or 1mM of the indicated compounds for 4 h. Metabolites are ordered from the highest to the lowest fold-change values in the MOG condition.

b) Amounts of the TCA intermediates fumarate and malate in HCC1569-EV or HCC1569-MCT2 cells treated with 1mM of the indicated compounds for 4 h. Significance tested by multiple t-tests with Holm-Sidak correction for multiple comparisons. (n=4 independent wells).

c) Labelling from [U-^{13}C]-Glutamine in HCC1569-EV or HCC1569-MCT2 cells after 4 h of incubation with 1 mM of the indicated analogues. M+4 citrate is derived from oxidative use of glutamine carbons through the TCA cycle, whereas m+5 is formed as a result of reductive carboxylation. Significance tested by multiple t-tests with Holm-Sidak correction for multiple comparisons. (n=4 independent wells).

d) Western blot showing protein levels of HIF1α and the mTORC1 kinase substrate S6K (total and phosphorylated at Thr389) in lysates of MCF7 cells starved of amino acids for 3 h in the presence or absence of the PHD inhibitor FG4592 (50 µM) or vehicle (DMSO), and subsequently stimulated with amino acids +/- 50 µM FG4592. Data show separate blots that were developed and exposed simultaneously.

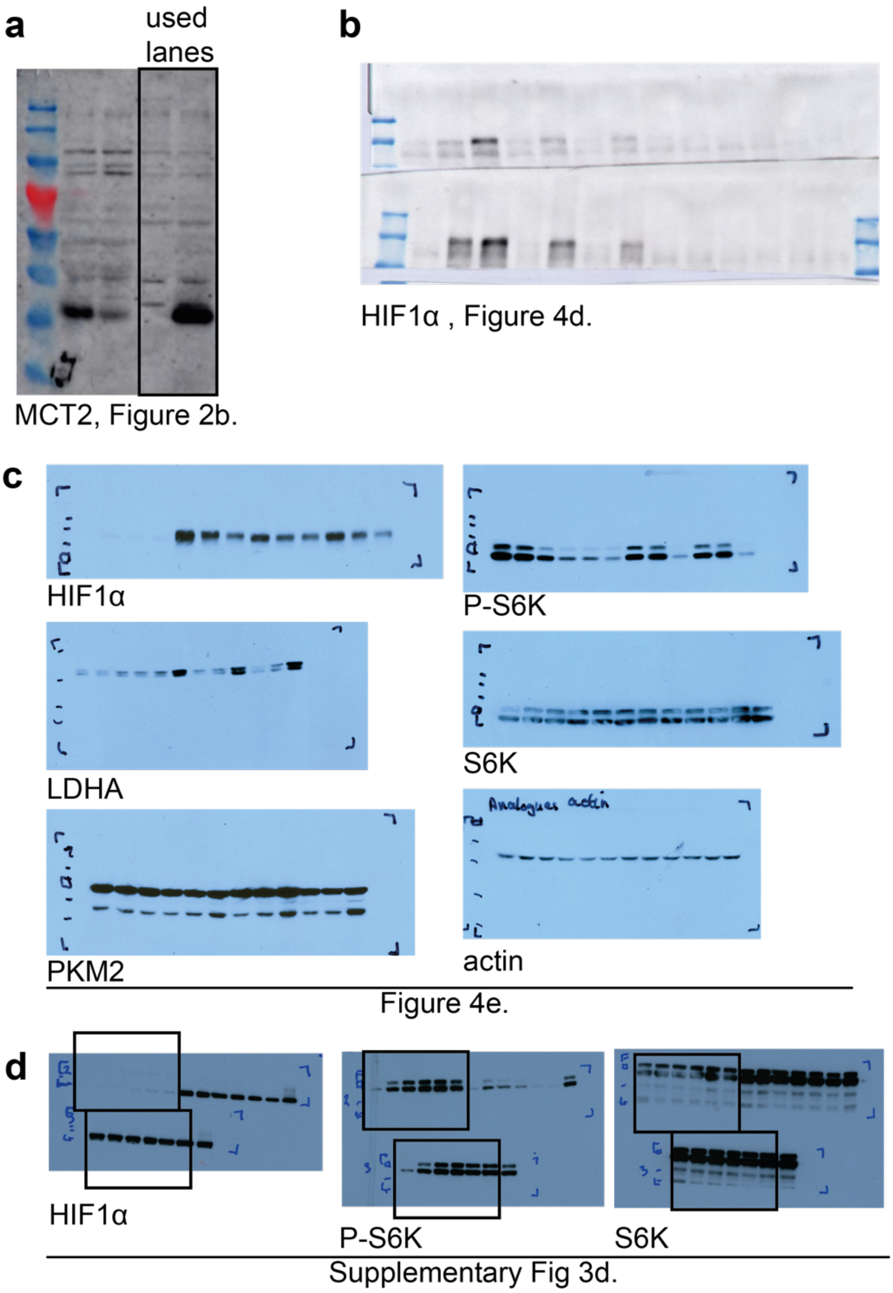

Supplementary Figure 4

a) Full scan western blots from Figure 2b.
b) Full scan western blots from Figure 4d.
c) Full scan western blots from Figure 4e.
d) Full scan western blots from Supplementary Figure 3d.

Supplementary Table 1

Mass spectrometry parameters used throughout this study for detection of MOG and MOG analogues by LC-MS.

Compound Name/Number	*m/z* (negative mode)	Fragments	HCD
DMOG	174.0408	-	
NOG	146.0095	74.0245	35
**1** (MOG)	160.0254	84.009, 59.013, 72.993, 100.004	35
**2**	174.0405	74.024, 84.009, 100.004	30
**3** (IPOG)	188.0567	74.024, 84.009, 100.004, 85.029	30
**4**	174.0405	98.025, 114.020, 72.993, 59.013	30
**5**	174.0405	98.025, 114.020, 72.993, 59.013	30
**6**	188.0559	102.0559, 128.0354	30
**7**	144.0302	99.926	35
**8**	169.0255	68.014, 82.029	30
**9**	236.0171	135.0058,114.9996, 164.0323	30
**10**	184.0358	89.0429, 68.9955	30
