# Supplementary material for: MOG analogues to explore the MCT2 pharmacophore, α-ketoglutarate biology and cellular effects of N-oxalylglycine
Source: Commun Biol. 2022 Aug 26;5:877. doi: 10.1038/s42003-022-03805-y (PMC9418262; doi:10.1038/s42003-022-03805-y)
Supplement: Supplementary file 3 — Description of Additional Supplementary Files [file 42003_2022_3805_MOESM3_ESM.pdf]

Description of Additional Supplementary Files

File name: Supplementary Data 1
Description: The source data underlying graphs shown in this study.
