# Supplementary material for: MOG analogues to explore the MCT2 pharmacophore, α-ketoglutarate biology and cellular effects of N-oxalylglycine
Source: Commun Biol. 2022 Aug 26;5:877. doi: 10.1038/s42003-022-03805-y (PMC9418262; doi:10.1038/s42003-022-03805-y)
Supplement: Supplementary file 5 — Reporting Summary [file 42003_2022_3805_MOESM5_ESM.pdf]

nature research

Corresponding author(s): Dimitrios Anastasiou

Last updated by author(s): 04.08.2022

Reporting Summary

Nature Research wishes to improve the reproducibility of the work that we publish. This form provides structure for consistency and transparency in reporting. For further information on Nature Research policies, see our Editorial Policies and the Editorial Policy Checklist.

Statistics

For all statistical analyses, confirm that the following items are present in the figure legend, table legend, main text, or Methods section.

n/a	Confirmed	
☐	☒	The exact sample size (n) for each experimental group/condition, given as a discrete number and unit of measurement
☐	☒	A statement on whether measurements were taken from distinct samples or whether the same sample was measured repeatedly
☐	☒	The statistical test(s) used AND whether they are one- or two-sided<br>*Only common tests should be described solely by name; describe more complex techniques in the Methods section.*
☐	☒	A description of all covariates tested
☐	☒	A description of any assumptions or corrections, such as tests of normality and adjustment for multiple comparisons
☐	☒	A full description of the statistical parameters including central tendency (e.g. means) or other basic estimates (e.g. regression coefficient) AND variation (e.g. standard deviation) or associated estimates of uncertainty (e.g. confidence intervals)
☐	☒	For null hypothesis testing, the test statistic (e.g. F, t, r) with confidence intervals, effect sizes, degrees of freedom and P value noted<br>*Give P values as exact values whenever suitable.*
☒	☐	For Bayesian analysis, information on the choice of priors and Markov chain Monte Carlo settings
☒	☐	For hierarchical and complex designs, identification of the appropriate level for tests and full reporting of outcomes
☐	☒	Estimates of effect sizes (e.g. Cohen's d, Pearson's r), indicating how they were calculated

Our web collection on statistics for biologists contains articles on many of the points above.

Software and code

Policy information about availability of computer code

Data collection	LC-MS instrument control: Xcalibur 3.0.63<br>Plate-reader acquisition software: Tecan iControl 1.11<br>Incucyte® Base Analysis Software
Data analysis	Graph plotting and statistical analysis: GraphPad Prism® 7.0b.<br>In-house scripting: RStudio version 1.0.136<br>GC-MS analysis: Agilent MassHunter Workstation software Quantitative Analysis Version B.06.00, or in house MANIC software (see materials and methods)<br>LC-MS analysis: Thermo Tracefinder 4.1 EFS

For manuscripts utilizing custom algorithms or software that are central to the research but not yet described in published literature, software must be made available to editors and reviewers. We strongly encourage code deposition in a community repository (e.g. GitHub). See the Nature Research guidelines for submitting code & software for further information.

Data

Policy information about availability of data

All manuscripts must include a data availability statement. This statement should provide the following information, where applicable:

- Accession codes, unique identifiers, or web links for publicly available datasets
- A list of figures that have associated raw data
- A description of any restrictions on data availability

The results shown in Supplementary Figure 1b are based upon data generated by the TCGA Research Network (https://www.cancer.gov/tcga). The source data

underlying graphs shown in this study are provided in Supplementary Data 1. Uncropped versions of the western blots used in this study (Fig. 2b, 4d, 4e, and Supplementary Fig. 3d) can be found in Supplementary Figure 4.

Field-specific reporting

Please select the one below that is the best fit for your research. If you are not sure, read the appropriate sections before making your selection.

☒ Life sciences ☐ Behavioural & social sciences ☐ Ecological, evolutionary & environmental sciences

For a reference copy of the document with all sections, see nature.com/documents/nr-reporting-summary-flat.pdf

Life sciences study design

All studies must disclose on these points even when the disclosure is negative.

Sample size	No sample size determination was performed. For metabolomics experiments, retrospective evaluation of data from pilot measurements indicated that 4 analytical replicates sufficed to yield statistical power that is acceptable for the value (<0.05) used in this manuscript to define experimental results as significant.
Data exclusions	N/A
Replication	All experimental findings presented in this study were reproducible based on independent replicate measurements as described in detail in the corresponding figure legends.
Randomization	For metabolomics analyses by GC-MS, samples were randomised using the relevant utility within the Agilent Chemstation software, and, for LC-MS analyses, randomisation was done using a random number generator in Microsoft Excel.
Blinding	No blinding was used

Reporting for specific materials, systems and methods

We require information from authors about some types of materials, experimental systems and methods used in many studies. Here, indicate whether each material, system or method listed is relevant to your study. If you are not sure if a list item applies to your research, read the appropriate section before selecting a response.

Materials & experimental systems

n/a	Involved in the study	
☐	☒	Antibodies
☐	☒	Eukaryotic cell lines
☒	☐	Palaeontology and archaeology
☒	☐	Animals and other organisms
☒	☐	Human research participants
☒	☐	Clinical data
☒	☐	Dual use research of concern

Methods

n/a	Involved in the study	
☒	☐	ChIP-seq
☒	☐	Flow cytometry
☒	☐	MRI-based neuroimaging

Antibodies

Antibodies used	Primary antibodies used were obtained from Cell Signalling Technology: P-S6 kinase #9234 1:1000, S6 kinase #2708 1:2000, LDHA: #2012 1:1000, PKM2 #3198 1:1000; Sigma: β-actin A5316 1:1000; BD Biosciences: HIF1α #610959 1:250; Rabbit-anti-MCT2 1:500 (generated by the Anastasiou lab). Secondary antibodies were goat anti-rabbit or anti-mouse IgG from Millipore (#AP132P, #AP127P respectively).
Validation	All commercial antibodies were validated as stated in the manufacturers' data sheets. Anti-MCT2 was validated using knock-down and over-expression cell lines (over-expression shown in manuscript).

Eukaryotic cell lines

Policy information about cell lines

Cell line source(s)	All cell lines were obtained from the American Type Culture Collection (ATCC, Manassas, VA, USA).
Authentication	Cell identity was confirmed by short tandem repeat (STR) profiling by The Francis Crick Institute Cell Services Science Technology Platform.

Mycoplasma contamination	All cell lines were tested mycoplasma-free by The Francis Crick Institute Cell Services Science Technology Platform.
Commonly misidentified lines (See ICLAC register)	No commonly misidentified cell lines were used.
